# Bioactivity-Guided Isolation of Flavone Glycoside from *Terminalia catappa*: Evaluating Anti-MRSA and Anti-Dermatophytic Potential

**DOI:** 10.3390/molecules30173595

**Published:** 2025-09-03

**Authors:** Tumakuru Nataraj Sowmya, Doddahosuru Mahadevappa Gurudatt, Koteshwar Anandrao Raveesha

**Affiliations:** 1 Department of Biotechnology and Bioinformatics, School of Life Sciences, JSS Academy of Higher Education and Research, Shivaratreeshwara Nagara, Mysuru 570015, Karnataka, India; 2 Center for Innovative Studies in Herbal Drug Technology, Department of Studies in Botany, University of Mysore, Manasagangotri, Mysuru 570006, Karnataka, India; karaveesha@gmail.com; 3 Department of Studies in Organic Chemistry, University of Mysore, Manasagangotri, Mysuru 570006, Karnataka, India; shivamma.guru@gmail.com; 4 School of Life Sciences, JSS Academy of Higher Education and Research, Shivaratreeshwara Nagara, Mysuru 570015, Karnataka, India

**Keywords:** flavonoids, apigenin, Combretaceae, *Terminalia* spp., MRSA, antimicrobial activity, TLC–bioautography, NMR analysis, global antimicrobial surveillance system

## Abstract

Antibiotic resistance is one of the major threats to public health in the twenty-first century. In this line of work, plants represent a priceless source of antimicrobial compounds since they house chemically different metabolites with a wide range of therapeutic applications. This study reports the bioactivity-guided fractionation, characterization, and evaluation of the efficacy of antimicrobial compounds from leaf acetone extracts of the traditional medicinal plant *Terminalia catappa* against bacterial clinical isolates and dermatophytes. The acetone extract of *T. catappa* was subjected to column chromatography for the separation and purification of the phytocompounds. The fractions were analyzed using a thin-layer chromatography–bioautography assay to detect the antimicrobial potency of the eluted compounds. The efficacy of the antimicrobial compounds was evaluated by the minimum inhibitory concentration, minimum bactericidal concentration, and minimum fungicidal concentration. Spectral characterization and structure elucidation of the compound were also achieved. The leaf acetone extract, when subjected to gradient elution by column chromatography, resulted in eight fractions. The fraction Fr-2 was subjected to thin-layer chromatographic elution, which resulted in the elution of phytocompound with R*f* value of 0.50 and the phytocompound exhibited antimicrobial activity in the TLC–bioautography assay, and it was isolated in pure form and confirmed as Apigenin 7-O-ß-D-glucopyranoside. The compound exhibited significant inhibition of the clinical isolate *Staphylococcus aureus* and Methicillin-resistant *Staphylococcus aureus* 1503 at 9.5 µg/mL. Dermatophytes, viz., *Microsporum gypseum* and *Microsporum canis*, were inhibited at 312 µg/mL. The present study successfully achieved the bioactivity-guided isolation and characterization of the flavone compound Apigenin 7-O-ß-D-glucopyranoside from *T. catappa*. Furthermore, the plant *T. catappa* represents a promising candidate for the exploration of antimicrobial compounds that could serve as potential plant-derived lead molecules for antimicrobial agents.

## 1. Introduction

The multidrug resistance (MDR) problem in the microbial world is a major challenge in treating infectious diseases [1]. With over 17 million fatalities annually, microbial infections continue to be a major worldwide health concern. Treatment failures are sharply rising as a result of the rise in MDR microorganisms [2]. Extended-Spectrum Beta-lactamases, Methicillin-resistant *Staphylococcus aureus*, Vancomycin-resistant *Staphylococcus aureus*, multidrug-resistant *Streptococcus pneumoniae*, Carbapenem-resistant enterobacteriaceae, and multidrug-resistant *Acinetobacter baumanii* are some examples of bacteria that develop resistance to different classes of antibiotics [3]. A Centers for Disease Control and Prevention (CDC) report claims that antibiotic-resistant bacteria in the US cause approximately 2 million illnesses and 23,000 deaths annually. The antibiotic drug resistance problem is envisaged to outclass the threat of cancer in the near future [4]. The CDC has also categorized different drug-resistant bacteria and fungi into urgent, serious, and concerning, based on their pathogenicity and the need for developing new drugs [5]. Similarly, according to the WHO, the Global Antimicrobial Surveillance System (GLASS) revealed that 500,000 individuals in 22 countries had antibiotic-resistant bacterial infections. It is estimated that by 2050, there will be 300 million premature deaths and USD 100 trillion in losses to the global economy [6].

In addition to bacterial drug resistance, dermatophyte infections are becoming more and more resistant to common antifungal therapies [7], including resistance to drugs such as polyene macrolides (amphotericin B), 1,3-β-glucan synthase inhibitors (echinocandins), and azole derivatives (ketoconazole, fluconazole, voriconazole, itraconazole), which exists in *Candida* species, *Aspergillus* species, *Cryptococcus neoformans*, *Trichosporon beigelli*, and *Scopulariopsis* species [8]. The fact that there are only three classes of antifungal medications available to treat systemic fungal infections further increases the impact of fungal infections on human health. The drugs include polyenes, which bind to ergosterol in the fungal cell membrane and cause cell lysis; azoles, which target ergosterol biosynthesis; and echinocandins, which prevent the manufacture of fungal cell walls [9].

The occurrence of cross-resistance in bacteria and fungi, along with the ineffectiveness of the present classes of antimicrobial agents, raises a compelling need to identify and develop antimicrobial drugs. With the goal of tackling these antibiotic-resistant pathogen-mediated illnesses, bioactive phytochemicals have been identified as an alternative to traditional antibiotics. Since ancient times, medicinal plants have been utilized to treat and cure illnesses [10,11]. Several phytochemicals have shown their potential as antimicrobials by targeting key mechanisms of antimicrobial resistance, like targeting cell membrane proteins, biofilm formation, efflux pumps, and others. This represents an appealing strategy for combating drug-resistant microorganisms [12,13].

A recent study reviewed 81 plant-sourced compounds posing anti-MRSA activity and 11 phytocompounds exhibiting synergistic anti-MRSA activity [10]. Various reports suggest the antimicrobial activity of different secondary metabolites from natural sources, like Quercetin, corilagin, Gallotannin, Myricetin, 4′,5′-*O*-decaffeoyl acetic acid, Luteolin-7-*O*-glucoside, and Epigallocatechin, such as inducing efflux pump inhibition, blocking DNA synthesis, inhibiting ATPase activity, targeting MFS transporters, and interacting with the GyrB protein [14,15,16,17].

TLC–bioautography is a sensitive and cost-effective technique that identifies the antimicrobial compounds in a mixture of plant extracts. This technique provides preliminary information on the antimicrobial nature of the separated phytocompounds and expedites the rapid and target-directed isolation of active principles, which aids in the drug discovery process [18].

Considering the reported potentiating effects of phytocompounds, the present study reports the bioactivity-guided isolation of antimicrobial compounds from the traditional medicinal plant *Terminalia catappa* L., which belongs to the family Combretaceae and is commonly known as tropical almond, Malabar almond, and Indian almond [19]. Traditionally, it is used to treat a variety of illnesses, including gastritis, pyrosis, diarrhea, dermatitis, headaches, leprosy, and urinary tract infections [20]. The leaves, fruit, and bark of the tree are ethnomedicinally used. In Southeast Asian countries, the bark is used to treat dysentery, while its root bark is used to treat thrush, bilious fever, and diarrhea. The fruit kernel, along with beeswax, is used to treat putrid exudation and bloody feces [21,22].

Reports suggest that the plant is known to possess many biological activities, like antioxidant, hepatoprotective, anti-inflammatory, antidepressant, antifungal, chemopreventive, antibacterial, and wound healing properties [23,24,25]. According to earlier phytochemical analysis of *T. catappa* leaf extracts, the species is mostly composed of a phenolic class of metabolites, which includes flavonoids, triterpenoids, and hydrolyzable tannin [26]. Detailed phytochemical investigations revealed the presence of compounds like punicalagin, punicalin, terflavin A and B, chebulic acid, tergallagin, chebulagic acid, geraniin, tercatain, coumaric acid, arjunolic acid, terminolic acid, corilagin, catappanin, geranin, Tergallagin, Catechin, and vitexin [27,28]. Our previous studies suggested that the crude and partially purified extract of *T. catappa* was rich in polyphenols and demonstrated promising antibacterial and antifungal activities [29]. The antimicrobial realm of study is moving quickly toward the identification of antimicrobial phytochemicals, understanding their modes of action, and incorporating them into potential cures and treatments [30]. In this regard, the data presented about the bioactive-guided isolation of the antimicrobial molecules from the plant *T. catappa* is limited, and only the activity of the crude extracts is reported. Hence, with this aim, the present work attempts the isolation and identification of the antimicrobial molecule by bioactive-guided isolation, which could add value and provide preliminary information in the wake of discovering antimicrobial lead molecules from the plant, with special reference to multidrug-resistant *Staphylococcus aureus strains* and dermatophytes.

## 2. Results

### 2.1. Column Chromatography and the TLC Profile of Isolated Fractions

**Figure 1:**
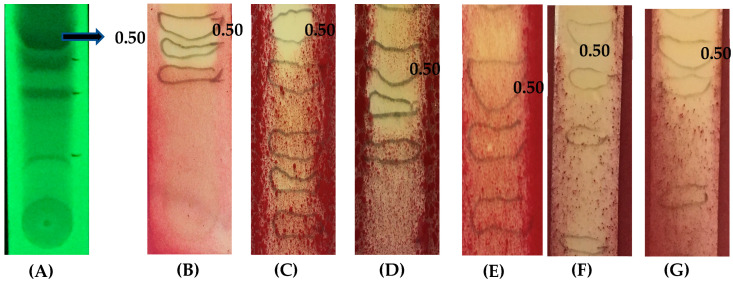
(**A**) TLC profile of Fr-2, (**B**–**G**) Agar overlay bioautography of the eluted phytocompounds from Fr-2 depicting antibacterial activity by exhibiting a clear zone of inhibition at R*f* 0.50. (**B**) *S. aureus* (clinical isolate), (**C**) *S. aureus* (MTCC 7443), (**D**) *S. typhi* (MTCC 733), (**E**) *P. vulgaris* (clinical isolate), (**F**) MRSA 1503, and (**G**) MRSA 1007.

The crude leaf acetone extract of *T. catappa* was fractionated by column chromatography to yield 154 fractions. The fractions exhibiting the same TLC profile were pooled and grouped into eight fractions, viz., Fr-1 (1–18), Fr-2 (27–45), Fr-3 (46–54), Fr-4 (55–72), Fr-5 (73–87), Fr-6 (88–100), Fr-7 (101–124), and Fr-8 (125–154). Among the eight fractions, fraction-2 (Fr-2) was subjected to TLC separation and exhibited clear separation of phytocompound with R*f* value 0.50 as shown in Figure 1A.

### 2.2. Agar Overlay Bioautography of Eluted Compound from Fraction 2

The separated phytocompound from Fr-2 were subjected to Agar overlay bioautography to assess the antimicrobial property. The band with R*f* value, 0.50 exhibited antimicrobial property by showing a clear halo zone and it was subjected to further isolation and purification. 

### 2.3. Agar Overlay Bioautography of Eluted Compounds from Fraction 2 Against Test Bacteria

The separated phytocompound from Fr-2 were subjected to Agar overlay bioautography to assess their antibacterial property. The band with *R*f value of 0.50 showed significant inhibition of all the test bacteria (Figure 1B–G). The most sensitive were the clinical isolate *S. aureus*, MRSA 1503, and MRSA 1007. Among the MTCC strains tested, *S. typhi* (MTCC 733) and *S. aureus* (MTCC 7443) were inhibited. The clinical isolate *P. vulgaris* showed minimum inhibition.

### 2.4. Agar Overlay Bioautography of Eluted Compounds from Fraction 2 Against Dermatophytes

**Figure 2:**
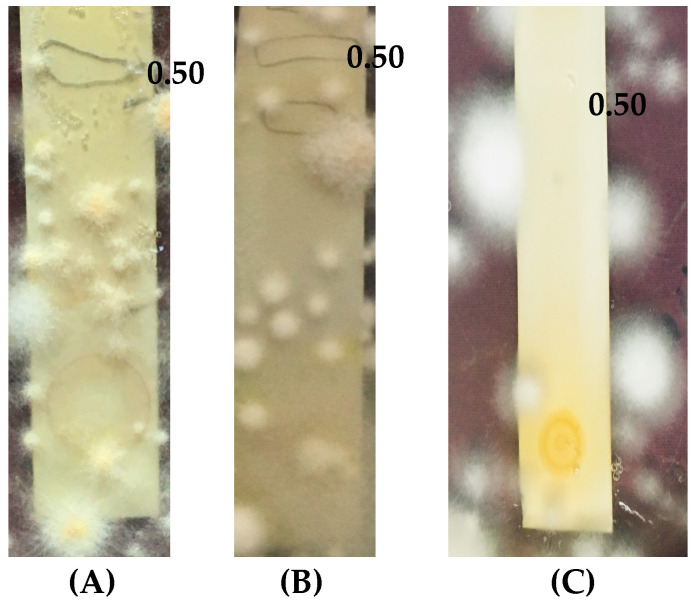
(**A**–**C**): Agar overlay bioautography of the eluted phytocompound/s with an R*f* value of 0.50, depicting anti-dermatophytic activity by exhibiting a clear zone of inhibition. (**A**) *M. canis*, (**B**) *M. gypseum*, and (**C**) *T. rubrum*.

The TLC-separated band from Fr-2 with R*f* value of 0.50 was subjected to agar overlay bioautography to assess the anti-dermatophytic potency. The band was shown to inhibit the dermatophytes, viz., *M. gypseum*, *T. rubrum*, and *M. canis* (Figure 2).

### 2.5. Isolation of the Antimicrobial Compound by Preparative Thin-Layer Chromatography

The band with an R*f* value of 0.50 that exhibited strong antimicrobial activity against the test bacteria and fungi was subjected to preparative thin-layer chromatography to isolate the compound in pure form. The eluted band was scraped, dissolved in methanol, and filtered. Further, it was stored at 4 °C in a screw-capped bottle for further characterization and evaluation.

### 2.6. Characterization and Structure Elucidation of the Antimicrobial Compound

#### 2.6.1. HRLCMS Profile of the Antimicrobial Compound

**Figure 3:**
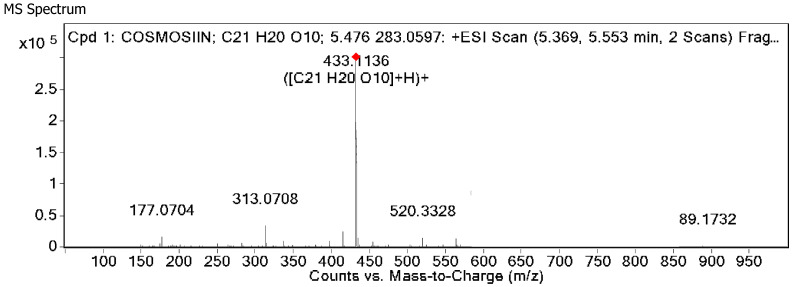
HRLCMS profile of Apigenin 7-O-ß-D-glucopyranoside exhibiting mass and characteristic fragments.

The LC-Q-TOF-MS/MS analysis of the active band with an R*f* value of 0.50 in positive ionization mode exhibited a peak with a retention time (Rt) of 5.83. The mass spectral analysis revealed the molecular weight of the compound to be 432.105, i.e., *m*/*z* 433.11 [M + H]^+^, with a parent fragment ion at 433.11 (Figure 3). The MS/MS analysis revealed fragment ions at 283, 269, 313, and 312, which are typical for apigenin glucosides. The molecular formula deduced based on the HRLCMS data was C_21_H_20_O_10_.

#### 2.6.2. FT-IR Analysis of the Antimicrobial Compound

The FTIR spectra of Apigenin 7-O-ß-D-glucopyranoside exhibited absorption bands at 3446.71 cm^−1^, arising from OH stretching. The peak at 3421.50 cm^−1^ may be attributed to the stretching vibration of aromatic C-H. There was a saturated hydrocarbon peak at 2924.52 cm^−1^.

**Figure 4:**
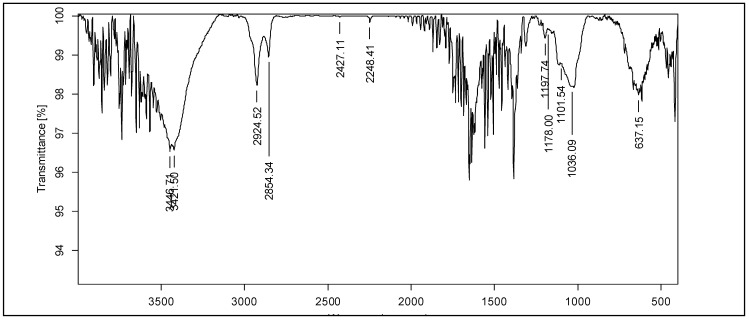
FTIR characterization of Apigenin 7-O-ß-D-glucopyranoside showing characteristic functional groups.

The absorption band at 2854.34 cm^−1^ corresponds to C-H stretching. The peak at 2248 cm^−1^ is due to the C triple bond N. 1652.19, and the 1622 cm^−1^ peak confirms the presence of the C=O functional group. The peaks at 1101.54 and 1036.09 cm^−1^ may be attributed to glycosidic C-O bonds, which are characteristic of apigenin glucoside. The absorption peak found at 1178 cm^−1^ may be attributed to the presence of carboxylic groups. The 637.15 cm^−1^ absorption is due to the O-H bend, and the 1197.74 cm^−1^ absorption is due to the C-O stretch. The spectra had characteristic absorption bands for apigenin glucoside (Figure 4).

#### 2.6.3. ^1^HNMR Analysis of Apigenin 7-O-ß-D-Glucopyranoside

**Figure 5:**
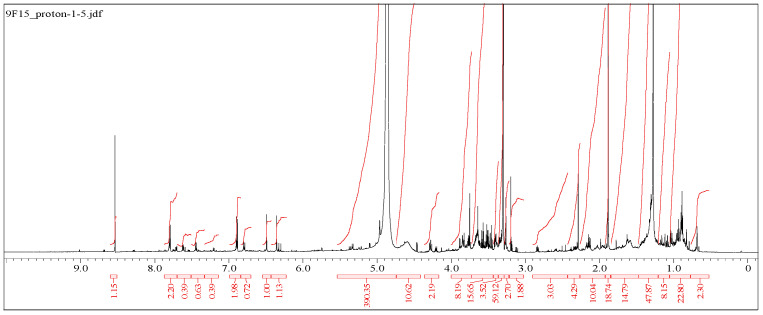
NMR profile of Apigenin 7-O-ß-D-glucopyranoside.

**Figure 6:**
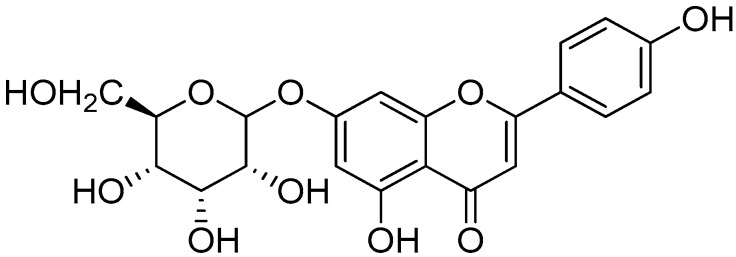
Structure of Apigenin 7-O-ß-D-glucopyranoside.

5-hydroxy-2-(4-hydroxyphenyl)-7-(((3R,4R,5S,6R)-3,4,5-trihydroxy-6-(hydroxymethyl)tetrahydro-2*H*-pyran-2-yl)oxy)-4*H*-chromen-4-one: C_21_H_20_O_10_ (Apigenin 7-O-ß-D-glucopyranoside, 4):^1^H-NMR (600 MHz, CH_3_OH D4): The pyranose ring appears at *δ* 3.30–3.54 (m, 3H, pyranose sugar protons), 3.60 (q, 1H), which is a quartet (q) with an integration value of 1H. A hydroxyl group (OH) signal appearing at δH 3.66 ppm (s, 3H, -OH) is typical of protons attached to a carbon atom directly adjacent to a hydroxyl group (CH-OH). A doublet at 3.68 ppm, with an integration of 2H and a chemical shift around 3.68 ppm (d, 2H, -CH_2_), indicates two protons of a methylene group (-CH_2_). A chemical shift of 3.72 ppm, with a singlet (s) and integration of 1H, indicates a proton on a methylene group (-CH_2_-) directly attached to an oxygen and a hydroxyl group (-CH_2_OH). This signal suggests the presence of an alcohol group. A signal at 5.28 ppm (d, 1H) indicates a proton that is deshielded and coupled to one neighboring proton. The “d” (doublet) indicates that the signal splits into two peaks because of the presence of one neighboring proton. A singlet (s) signal at 5.88 ppm, with the integration of 2H (indicating 2 protons), is often assigned to a phenolic -OH group. This region is relatively common for phenolic protons and typically appears as a singlet because of the lack of nearby coupling interactions. The 6.20–7.92 (m, 7H) range falls within the aromatic region of a proton NMR spectrum, which is characteristic of aromatic protons (Figure 5). After NMR analysis, the structure of Apigenin 7-O-ß-D-glucopyranoside was deduced (Figure 6). 

### 2.7. Efficacy of Apigenin 7-O-ß-D-Glucopyranoside Evaluated by Minimum Inhibitory Concentration and Minimum Bactericidal Concentration

#### 2.7.1. Antibacterial Activity of Apigenin 7-O-ß-D-Glucopyranoside

**Figure 7:**
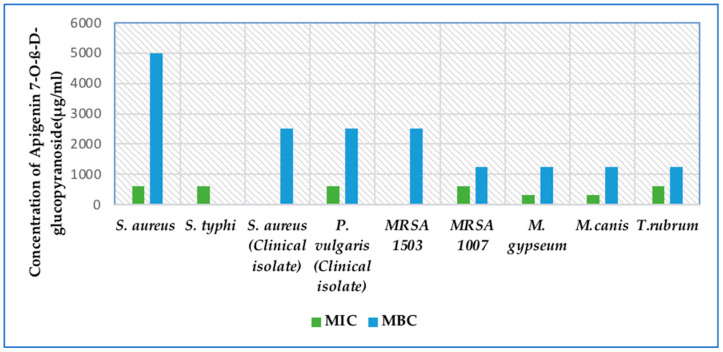
Graph depicting the efficacy of Apigenin 7-O-ß-D-glucopyranoside, evaluated by the minimum inhibitory concentration, minimum bactericidal, and fungicidal concentration against test bacteria and dermatophytes.

MIC assays revealed that the most sensitive organisms that were inhibited by Apigenin 7-O-ß-D-glucopyranoside were the clinical isolate *S. aureus* and MRSA 1503, with an inhibitory concentration of 9.5 µg/mL. *S. aureus* (MTCC 7443) and *S. typhi* (MTCC 733) were inhibited at 625 µg/mL. MRSA 1007 and the clinical isolate *P. vulgaris* exhibited an MIC of 625 µg/mL (Table 1 and Figure 7).

The MBC ranged from 5000 to 1250 µg/mL for all the test bacteria. Although the clinical isolate *S. aureus* and MRSA 1503 exhibited low MIC values (9.5 µg/mL), the compound did not exhibit bactericidal properties at the test concentration. In contrast, at higher MIC values (625 µg/mL), the compound exhibited bactericidal effects against MRSA 1007 and the clinical isolate *P. vulgaris* (Table 1 and Figure 7).

#### 2.7.2. Antifungal Activity of Apigenin 7-O-ß-D-Glucopyranoside Against Dermatophytes

Apigenin 7-O-ß-D-glucopyranoside had considerably better activity against the fungi in terms of fungicidal activity in contrast to bacteria (Table 1 and Figure 7). *M. gypseum* and *M. canis* exhibited an MIC of 312 µg/mL. *T. rubrum* exhibited an MIC of 625 µg/mL. The reported MFC was 1250 µg/mL. The compound exhibited fungicidal activity against all the test fungi at the test concentration (Table 1 and Figure 7).

## 3. Discussion

Bacteria have always been a problem for humans, and several drugs have been produced in order to treat them. However, the proteins and genetic makeup of bacteria continue to change and regenerate, resulting in increased resistance to drugs [31]. Antibiotics are now considered a global danger by the WHO, and the Global Antimicrobial Resistance and Use Surveillance System (GLASS) Report from 2021 shows that antimicrobial-resistant bacteria are mostly accountable for the precipitous increase in bacterial infections. The increasingly restricted availability of effective medicines to combat the problems caused by bacteria sparked interest in the development of novel antibacterial agents [32]. The present study systematically achieves the bio-guided fractionation of an antimicrobial compound from the plant *T. catappa*. Previously, different secondary metabolites (like flavonoids and terpenoids) were identified in the polar extracts of *T. catappa* [27,28,33,34]. The preliminary results of our previous work suggest that the plant *T. catappa* is a rich source of polyphenols, including terpenoids and tannins [29].

In the present research, we report the bioactivity-guided isolation of Apigenin 7-O-ß-D-glucopyranoside from the leaf acetone extract of *T. catappa*. UHPLC-MS/MS fragment analysis of the active band confirmed that the isolated lead compound was Apigenin 7-O-ß-D-glucopyranoside, belonging to the flavone group, which corroborates the previous literature [35,36]. FT-IR analysis of the compound was confirmed by the presence of characteristic absorbance for flavonoids at 3825 cm^−1^–2169 cm^−1^ for O-H functional groups [37] and 1036 cm^−1^ for the glycosidic moiety [38,39]; 1652 cm^−1^ and 1622 cm^−1^ confirmed the presence of C-O groups [40]. NMR spectra depicted the presence of an apigenin nucleus and attachment of glucose residues [41,42,43]. The antibacterial investigation revealed that Apigenin 7-O-ß-D-glucopyranoside significantly inhibited MRSA 1503 (9.5 µg/mL) and the clinical isolate *S. aureus* (9.5 µg/mL). Interestingly, Gram-negative *S. typhi* (MTCC 733) also exhibited susceptibility to Apigenin 7-O-ß-D-glucopyranoside.

Apigenin 7-O-ß-D-glucopyranoside also exhibited significant growth inhibition of dermatophytes. The present study reports significant activity against Gram-positive compared to Gram-negative bacteria. The anti-dermatophytic activity obtained also corroborates previous research [44,45]. Previous research on different extracts of T. catappa suggests the presence of hydrolyzable tannins, flavonoid glycosides, and gallic acid, which exhibited antifungal activity [46].

The antibacterial activities of flavonoids are closely related to their structural characteristics. Flavones, in particular, are known to exhibit several biological activities, namely, anti-inflammatory, anti-allergic, antiviral, antioxidant, antitumor, neuroprotective, antimicrobial, and cardioprotective [47]. It has been shown that the 5,7-OH groups of the A-ring and the 4′-OH group of the B-ring in apigenin were important in reversing antimicrobial resistance [48]. Investigations by other researchers suggest that apigenin glycoside has stronger antibacterial efficacy compared to aglycone apigenin. This property may be due to the increased solubility and amphiphilicity of glycosylated plant metabolites, which enhance their membrane transport [49]. The present work is supported by the fact that the isolated molecule was water soluble and had significant anti-MRSA activity. The antibacterial research on apigenin and other related flavonoids shows that the activity ranged from 250 to 1000 µg/mL. This supports the fact that flavonoids generally have higher antimicrobial activity [50]. The differences in the values of the MIC reported in the present investigation compared to the previous research may be due to strain sensitivity, since different strains of bacteria and fungi will exhibit different sensitivities.

Flavonoids, in general, are suggested to possess various biological activities, like antioxidant, antiviral, antitumor, antibacterial, antiaging, and other pharmacological effects [51]. Flavonoids’ antimicrobial properties can be influenced by the presence of an aromatic ring, the sugar moiety, or the number of hydroxyl and methoxyl groups, which alter membrane permeability and subsequent affinity to the binding sites in bacteria [52].

Another fact that alters the antimicrobial activity of apigenin and its glycosides is the placement of the hydroxyl group in the A-ring and the absence of an OH group on the B-ring. This is very important for the antibacterial activity of flavonoids because these groups have a strong affinity to the polar phospholipid region on the bacterial membrane [53]. Structure–activity relationship studies have been conducted to study the antibacterial activity on MRSA, and the results showed that the OH group on the B-ring of the flavone skeleton exhibited anti-MRSA activity, which ranged from 0.5 to 2 µg/mL. The hydroxy group on the A-ring and B-ring favored anti-MRSA activity [54]. The results obtained in our study are in line with the abovementioned research, where the OH groups can be found in the A-ring (5′-position) and B-ring (4′-position) of the flavone skeleton. This could be the reason for the inhibition of MRSA 1503 and other test bacteria at lower MIC values. The mechanism of action of flavonoids suggests that they can disrupt the membrane structure by disordering and disorienting membrane lipids, resulting in membrane leakage. Flavonoids also interfere with the cell cycle, cell division, and DNA and RNA synthesis, thereby acting as antifungals [55]. Several studies allow us to state that the primary ways in which flavonoids work against bacteria are by inhibiting the production of nucleic acids, inhibiting the function of the cytoplasmic membrane by influencing the creation of biofilms, porins, permeability, and interacting with certain important enzymes [50]. The number and position of the methoxy and hydroxyl groups and the principal structural moieties in the lead molecule have a great impact on the antimicrobial properties [56]. However, there are reports suggesting the higher antimicrobial activity of flavonoids, and different research groups have recorded a varied range of antimicrobial activity of apigenin and other related flavonoids against both Gram-positive and Gram-negative bacteria. The discrepancies in the sensitivities of the organisms towards the lead molecule could also be attributed to the concentration used in this study, the solubility of the active compound, and the choice of solvent used in the extraction and separation methods [50,57]. But since the pharmaceutical use of flavonoids is frequently addressed, it is important to come to a consensus regarding how to interpret flavonoid MIC values [57].

## 4. Materials and Methods

### 4.1. Chemicals and Microorganisms 

Human pathogenic bacteria were obtained from the Microbial Type Culture Collection (MTCC), Chandigarh, India, viz., *Salmonella typhi* (MTCC 733) and *Staphylococcus aureus* (MTCC 7443). Clinical isolates *Staphylococcus aureus*, *Proteus vulgaris*, MRSA 1503, and MRSA 1007 were kindly provided by the Microbiology Department, Government Medical College, Mysuru, Karnataka, India. The pathogens were maintained on Muller–Hinton agar (Himedia, Bangalore, India). The bacteria were resuscitated by sub-culturing from stock onto Muller–Hinton agar plates, followed by incubation overnight for their optimum growth. The dermatophytes used in this study were procured as freeze-dried cultures from the MTCC, Chandigarh, India. *Microsporum canis* (MTCC 2820), *Microsporum gypseum* (MTCC 2830), and *Trichophyton rubrum* (MTCC 296) were maintained on Sabouraud dextrose agar (Himedia, Bangalore, India). Ciprofloxacin and Miconazole (Himedia, Bangalore, India) were used as antibacterial and antifungal agents, respectively. TLC precoated sheets (Merk silica gel F_254_, Bangalore, India) were used for TLC–bioautography assays.

### 4.2. Extraction and Purification

Healthy leaf material was collected, washed, and shade-dried. The plant material was powdered and subjected to Soxhlet extraction with polar and non-polar solvents. The extracts were concentrated to dryness and stored until further use. Our previous study investigated the bioactivity of different solvent extracts of *T. catappa*. During the course of the study, the acetone extract was subjected to disc diffusion assays, which showed significant inhibition of test microbes. Hence, the acetone extract was subjected to further isolation and purification of the antimicrobial compounds.

The acetone extract was subjected to silica gel column chromatography (200–400 mesh), with stepwise gradient elution, using ethyl acetate and methanol as the eluent system (10:0, 9:1, 8:2, 7:3, 6:4, 5:5, 4:6, 3:7, 2:8 and 0:10) to obtain 154 fractions of 12 mL, each with a flow rate of 1 mL/min. The fractions were subjected to TLC to check their TLC profile with ethyl acetate–methanol–water as the eluent system with different eluent ratios. The fractions containing the same TLC profile were combined to obtain a total of 8 fractions (Fr-1–Fr-8). Fr-1–Fr-8 were subjected to agar overlay bioautography to detect the antimicrobial compounds, and the fraction showing significant antimicrobial activity in the bioactivity-guided assay was subjected to further antimicrobial assays, characterization, and structural elucidation of the antimicrobial compound.

### 4.3. Detection of Antimicrobial Compounds by the Agar Overlay Bioautography Assay

Agar overlay bioautography was carried out by adopting the methodology of [58] with slight modifications. Fr.1–Fr.8 were subjected to TLC–bioautography by spotting the concentrated fraction on precoated silica gel plates (Merk Silica gel 60 F_254_; 20 × 20 cm), which were eluted with the ethyl acetate–methanol–water (6.2:0.7:0.5, *v*/*v*/*v*) solvent system. Among the 8 fractions, Fr.2 exhibited clear elution of compound/s and was subjected to agar overlay bioautography to detect the antimicrobial potency of the compound/s. The eluted band/s exhibiting antimicrobial activity was scraped from the plate, dissolved in a suitable solvent, filtered and used for further assays.

### 4.4. Characterization and Structure Elucidation of the Antimicrobial Compound

#### 4.4.1. High-Resolution LCMS Characterization of the Antimicrobial Compound

The isolated antimicrobial compound was subjected to HRLC-Q-TOF-MS/MS to determine its molecular weight. A Hypersil gold column (C18X 2.1 mm-3Micron) was used for chromatographic separation, which was performed on a UHPLC column Agilent (6550 ifunnel Q-TOF’s). Solvent A consisted of 0.1% formic acid in water, and Solvent B consisted of 90% acetonitrile, 10% water, and 0.1% formic acid. The injection volume was set at 5 µL, and the flow rate was adjusted to 0.3 mL/minute. Mass detection was performed using a Q-TOF mass spectrometer (Agilent Technologies, Santa Clara, CA, USA), which had AJS-ESI as an ion source with a scan range of 500–1000 M/Z. The capillary tension was set at 3500 V, and the gas flow was set at 13 L/min, with a 250 °C temperature. The sheath gas flow rate was 11 L/min at 300 °C. The nebulizer gas was set at a 35 psi gas flow pressure. Q-TOF data acquisition and the evaluation of mass spectrometry were carried out using the Agilent Metlin database.

#### 4.4.2. FT-IR Characterization of the Antimicrobial Compound

The presence of functional groups of the active principle/s was obtained using a 3000 Hyperion microscope with the vertex 80 FTIR system (Bruker, Bremen, Germany). The sample was dissolved in HPLC-grade methanol and encapsulated in KBr; the spectra were measured within a range of 4000–500 cm^−1^.

#### 4.4.3. ^1^HNMR Spectral Analysis of the Antimicrobial Compound

The NMR spectra of the isolated active principle were recorded on a 600 MHz NMR mass spectrometer (JOEL, Tokyo, Japan) with methanol D4 as the solvent at 600 MHz. The data were expressed in parts per million.

### 4.5. Antimicrobial Efficacy of the Isolated Antimicrobial Compound

The MIC was established by employing a 96-well microtiter plate according to the CLSI M07-A9 for bacteria and the CLSI M38 A document for fungi [59,60]. Serial to two-fold dilution of the pure compound was conducted to obtain a concentration of 5000-9 μg/mL. The experimental setup included reference drug controls and a solvent as positive and negative controls, respectively. An aliquot of 0.5 McFarland standard inoculum was added to all the wells. Inhibition of bacterial growth was confirmed by adding 20 μL of an aqueous solution of 2,3,5-Triphenyl tertrazolium chloride (TTC) and re-incubating for 4–5 h. The colorless well was designated as the MIC. The MBC was ascertained by sub-culturing 10 μL of a test dilution from the lowest concentration well, followed by streaking on the previously sterilized and solidified MH agar, with overnight incubation. The well that gave no bacterial colony was designated as the MBC.

## 5. Conclusions

It is crucial for researchers to find natural compounds with antibacterial and antifungal drug-like phytocompounds in order to counteract antibiotic resistance. The present study systematically isolated and purified Apigenin 7-O-ß-D-glucopyranoside, a flavone glycoside from leaf extracts of *T. catappa*. Agar overlay TLC–bioautography is a convenient first-stage screening technique that helps to detect antimicrobial compounds. Apigenin 7-O-ß-D-glucopyranoside exhibited broad-spectrum antimicrobial activity by significantly inhibiting both bacteria and dermatophytes. It is significant to note that the compound exhibited anti-MRSA activity, which further confirms the antibacterial activity of flavonoids. The plant *T. catappa* is a rich source of flavonoids and terpenoids, which was proven in our previous studies. Thus, the plant could be further explored for other bioactive molecules, the mechanism of action involved, and the evaluation of toxicity studies to potentially develop antimicrobial drugs.

## Figures and Tables

**Table 1:** Table showing the efficacy of Apigenin 7-O-ß-D-glucopyranoside against human pathogenic bacteria and dermatophytes.

Test Organism/ Test Compound	*S. aureus* (MTCC 7443)	*S. typhi* (MTCC 733)	*S. aureus* (Clinical Isolate)	*P. vulgaris* (Clinical Isolate)	MRSA 1503	MRSA 1007	*M*. *gypseum*	*M. canis*	*T. rubrum*
**Apigenin 7-O-ß-D-glucopyranoside (µg/mL)**	625	625	9.5	625	9.5	625	312	312	625
**MBC/MFC**	5000	*	2500	2500	2500	1250	1250	1250	1250
**Effect**	Bacteriostatic	Effect not achieved	Bacteriostatic	Bactericidal	Bacteriostatic	Bactericidal	Fungicidal	Fungicidal	Fungicidal
**Ciprofloxacin** **(µg/mL)**	0.019	0.019	0.019	0.019	0.019	0.019	--	--	--
**Miconazole** **(µg/mL)**	--	--	--	--	--	--	0.019	0.019	0.019

Note: ‘*’ Asterisks indicate no MBC achieved at the test concentration; -- indicates not applicable.

## Data Availability

All the data available within the text.

## Abbreviations

The following abbreviations are used in this manuscript:

MDR	multidrug resistance
MRSA	Methicillin-resistant *Staphylococcus aureus*
CDC	Center for Disease Control and Prevention
GLASS	global antimicrobial surveillance system
CAGR	compound annual growth rate
WHO	World Health Organization
TLC	thin-layer chromatography
Rt	retention time
Rf	retention factor
MIC	minimum inhibitory concentration
MFC	minimum fungicidal concentration
MBC	minimum bactericidal concentration
TTC	2,3,5-Triphenyl tertrazolium chloride
